# Machine Learning-Based Framework for Pre-Impact Same-Level Fall and Fall-from-Height Detection in Construction Sites Using a Single Wearable Inertial Measurement Unit

**DOI:** 10.3390/bios15090618

**Published:** 2025-09-17

**Authors:** Oleksandr Yuhai, Yubin Cho, Joung Hwan Mun

**Affiliations:** Department of Bio-Mechatronic Engineering, College of Biotechnology and Bioengineering, Sungkyunkwan University, Suwon 16419, Republic of Korea; oleksandr@g.skku.edu (O.Y.); yubinc@g.skku.edu (Y.C.)

**Keywords:** pre-impact fall detection, wearable inertial measurement unit, ensemble feature selection, gradient-boosted decision trees, construction safety

## Abstract

Same-level-falls (SLFs) and falls-from-height (FFHs) remain major causes of severe injuries and fatalities on construction sites. Researchers are actively developing fall-prevention systems requiring accurate SLF and FFH detection in construction settings prone to false positives. In this study, a machine learning-based approach was established for accurate identification of SLF, FFH, and non-fall events using a single waist-mounted inertial measurement unit (IMU). A total of 48 participants executed 39 non-fall activities, 10 types of SLFs, and 8 types of FFHs, with a dummy used for falls exceeding 0.5 m. A two-stage feature extraction yielded 168 descriptors per data window, and an ensemble SHAP-PFI method selected the 153 most informative variables. The weighted XGBoost classifier, optimized via Bayesian techniques, outperformed other current boosting algorithms. Using 5-fold cross-validation, it achieved an average macro F1-score of 0.901 and macro Matthews correlation coefficient of 0.869, with a latency of 1.51 × 10^−3^ ms per window. Notably, the average lead times were 402 ms for SLFs and 640 ms for FFHs, surpassing the 130 ms inflation time required for wearable airbags. This pre-impact SLF and FFH detection approach delivers both rapid and precise detection, positioning it as a viable central component for wearable fall-prevention devices in fast-paced construction scenarios.

## 1. Introduction

Falls continue to pose the most significant safety risk on construction sites worldwide. Multiple large-scale epidemiological studies indicate that falls account for roughly half of serious injuries and nearly one-third of fatalities [1,2,3,4,5]. Recent research demonstrates that falls constitute 59.61% of all occupational accidents within the construction industry [6] and account for about 43.5% of fatal occupational incidents in the USA [7]. Several studies have reported a persistent increase in fall-related fatalities in recent years [6,7,8], highlighting that falls are both the primary and increasingly frequent cause of serious injuries and deaths in the construction industry.

All construction-site falls are generally categorized as same-level falls (SLFs) or falls-from-height (FFHs) [2,9]. SLFs most frequently result from ground-level hazards, including wet or uneven surfaces, debris, loose cables, or ice, each of which can diminish friction and abruptly disturb a worker’s balance, ultimately leading to a fall [10,11,12]. In contrast, FFHs typically stem from issues such as inappropriate ladder positioning, unguarded scaffold edges, unprotected roof openings, or structural failures in temporary work platforms, causing workers to fall to a substantially lower level [11,12]. SLFs represent nearly 65% of all non-fatal construction-related fall incidents, establishing them as the most prevalent source of non-fatal occupational injuries [10,13,14]. By contrast, FFHs are responsible for the highest proportion of fatalities, accounting for approximately 79% of all deaths related to construction falls [15]. Notably, a significant proportion of fatal falls have occurred from low heights—about 2 to 4.5 m—predominantly involving incidents from roofs, scaffolds, or ladders, with craniospinal trauma most frequently cited as the cause of death [12,15]. Accordingly, recent advances in safety and occupational health research underscore the critical need for comprehensive risk mitigation strategies that focus on proactive detection and prevention of both SLF and FFH events [9,10,11,12,13,14,15,16].

Targeted training campaigns have significantly increased construction workers’ awareness of fall-prevention [17,18,19]; OSHA’s stand-down initiative alone has trained over a million employees and led to improvements in on-site practices [19,20]. However, personal fall-arrest systems prevent only approximately 5% of fatal incidents [15], and their effectiveness can be reduced by risks such as suspension trauma, anchor failure, or the 4.6 m clearance needed for proper operation [15,21]. Since fall-related injuries and fatalities continue to increase [3,22,23], recent studies have explored the development of wearable airbags that protect the hips during SLFs [24,25,26,27,28], as well as mitigate the impact of FFHs from heights of 2–5 m [24,28,29,30,31,32]. The successful use of these devices requires not only rapid inflation mechanisms but also an algorithm capable of reliably detecting both SLF and FFH events, which needs to be actuated early enough to guarantee full inflation before impact [26,30,33]. Few studies specifically focus on pre-impact detection concerning SLFs [26,33,34,35] or FFHs [29,31,36,37,38,39], and currently, no approach incorporates both fall hazard types within a unified framework.

To accurately detect pre-impact SLFs or FFHs, most studies employ a small, lightweight wearable inertial measurement unit (IMU), offering cost-effectiveness, reduced power demands, and the potential for high-speed accelerometer and gyroscope data acquisition [26,29,31,33,35,37,38,39,40,41,42]. IMU-based pre-impact SLF or FFH detection techniques are primarily categorized into threshold-based and machine learning-based approaches [35,38]. Threshold-based methods typically involve predetermined thresholds derived from real-time accelerometer and gyroscope measurements—such as specifying limits for resultant acceleration or angular velocity—to distinguish between normal activities and fall incidents. While threshold-based approaches are straightforward and enable rapid data processing, they are limited by inherent factors, such as sensitivity to individual variability, reduced ability to separate falls from highly dynamic non-fall motions, and a propensity for false positives during demanding construction activities [31,35,43].

Due to these limitations, recent research has shifted toward machine learning-based approaches that utilize advanced pattern recognition techniques [35,39,40,41,42,43]. These approaches typically begin with IMU data collection, followed by comprehensive feature extraction processes, such as deriving sum magnitude vectors, calculating mean and maximum values, assessing signal energy, and extracting frequency-domain features from accelerometer and gyroscope signals; these features are subsequently input to classification algorithms designed to precisely identify pre-impact SLFs or FFHs [35,39,40]. Machine learning-based methods offer advantages such as adaptability to various user populations and activities, enhanced generalization capabilities, and increased detection accuracy relative to threshold-based approaches [35]. Nonetheless, these methods face several obstacles, including limited applicability to diverse activity ranges, significant computational demands, and the critical need for careful feature selection, since the inclusion of irrelevant or redundant features can negatively impact the effectiveness of the algorithm [44,45]. Notably, most pre-impact SLF algorithms were designed using datasets that simulate only the passive activities typical of older adults, which results in inadequate performance when these algorithms are exposed to the intense and unpredictable movements frequently encountered on construction sites [38,39,40,41,42,43]. Similarly, existing machine learning-based pre-impact FFH detection research rarely incorporates highly dynamic and physically demanding construction-specific activities, such as the use of high-vibration tools and jumping, into their datasets [31,38,39]. Such activities exhibit substantial overlap with fall-like kinematic signatures and may result in false-positive detections if not accurately classified; therefore, it is critical to determine and utilize features that enable machine learning algorithms to effectively differentiate overlapping motion patterns to reduce these misclassifications [31,38,46]. Nevertheless, most current studies consider only basic one-step features [35,39], including resultant magnitudes and statistical metrics, and generally lack in-depth evaluation of the individual and combined impact of proposed features on fall detection accuracy. To the best of our knowledge, there is an absence of comprehensive studies that classify typical construction site activities—including high-intensity motions that frequently trigger false alarms—as distinct from pre-impact SLF and FFH events. In addition, reliable detection of pre-impact SLFs presents further difficulties because the lead time—defined as the duration from detection activation to the onset of a fall impact [40,41,42]—must be longer than both the inflation period of the wearable airbag (typically around 130 ms [29,47]) and an additional safety margin. The literature shows that robust SLF detection demands a lead time greater than 130 ms to guarantee proper deployment before impact, whereas for FFHs, the lengthier free-fall periods naturally offer more extensive detection opportunities [38,40,42,47]. Therefore, current pre-impact fall detection techniques in construction primarily center on identifying SLF or FFH events during the free-fall stage, frequently neglecting comprehensive assessment of the algorithm’s processing latency, which can result in inadequate lead time—especially undermining the prompt detection and mitigation of SLFs by fall-prevention devices.

To address the limitations of current pre-impact fall detection methods, this study seeks to develop a machine learning-based approach that enables accurate classification of SLFs, FFHs, and non-fall activities within construction site environments using a single IMU-equipped sensing device. Construction sites present challenging and dynamic activity profiles, including high-vibration machinery operation and jumping, which can generate signals that closely resemble SLFs and FFHs, thereby complicating the classification process and increasing the risk of false positives, which must be minimized to improve the effectiveness of risk assessment and fall-prevention systems, such as wearable airbags. To accomplish this, a custom waist-attached IMU device was first designed for the collection of tri-axial accelerometer and gyroscope data. Next, a comprehensive experimental dataset was collected, which included instances from three class labels: non-fall events (comprising 16 activities of daily living (ADLs), 10 near-falls, and 13 construction-activity types), SLF events (10 variants, including slip and trip events specific to construction sites), and FFH events (8 categories, covering various fall modalities and heights up to 2 m). Third, a two-stage feature extraction method was employed: time-series features were extracted from the raw tri-axial accelerometer and angular velocity signals, which were then used to compute statistical descriptors within defined windows. An ensemble feature selection strategy was then implemented, where features selected by both SHAP-Select [48] and permutation feature importance (PFI) [49] were combined, resulting in a high-performing and robust feature set appropriate for complex, imbalanced, multi-class classification challenges. Finally, advanced weighted boosting machine learning algorithms [50] were evaluated to determine the optimal classifier, balancing the accuracy of multi-class classification with computational efficiency to ensure suitability for real-time risk assessment and fall-prevention devices, including wearable airbags. This study delivers a robust and efficient IMU-based algorithm capable of reliably detecting pre-impact SLF and FFH events in the challenging, dynamic conditions characteristic of construction sites.

## 2. Materials and Methods

### 2.1. Data Collection

#### 2.1.1. Subjects

A total of 48 healthy adults, including 30 men and 18 women, participated in this experiment. Participants’ ages ranged from 21 to 34 years (mean of 25.3 years). Body mass ranged from 45 to 85 kg (mean of 64.8 kg), while standing height was between 1.57 and 1.90 m (mean of 1.69 m). None of the individuals reported any neurological disorders, balance issues, or musculoskeletal injuries that could impact normal movement. Written informed consent was secured from all volunteers prior to participation, and the research protocol received approval from the Sungkyunkwan University Research Ethics Committee.

#### 2.1.2. Apparatus

**Figure 1:**
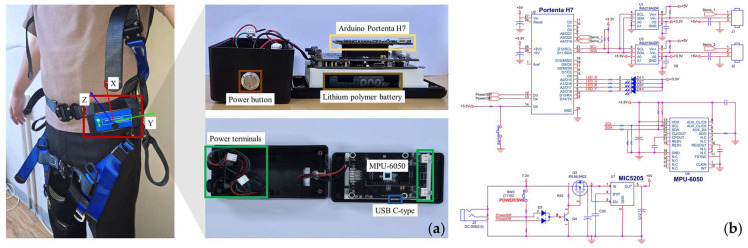
Pre-impact SLF and FFH detection device: (**a**) Sensing IMU device; (**b**) Electronic circuit diagram.

A custom single-sensor wearable device was developed using an IMU module (MPU-6050, JK Electronics, Seoul, Republic of Korea), which integrates a tri-axial accelerometer and gyroscope, along with an on-board 16-bit ADC for synchronous sampling. The accelerometer full-scale range was set to ±16 g, and the gyroscope full-scale range was set to ±2000 °/s. Sensor signals were sampled at 40 Hz, a rate selected to capture the approximately 20 Hz bandwidth of human motion with a margin, while minimizing noise, data volume, and power consumption. This choice is consistent with prior gait and activity-recognition studies, which report no significant accuracy gain above about 40 Hz [51,52,53]. Wireless transmission and on-board preprocessing were managed by a development board (Arduino Portenta H7, Arduino Holding, Monza, Italy), equipped with a dual-core microcontroller (STM32H747XI, STMicroelectronics, Geneva, Switzerland), 8 MB SDRAM, and 16 MB NOR flash memory. Bluetooth 5.1 connectivity on the board enabled low-latency data transfer to a workstation for live data collection. Power was provided by two 3.7 V/2000 mAh lithium-ion batteries (LB100BOX, Lightcom Corp., Seoul, South Korea). A C# Windows-forms application (HMSoft, JN Huamao Technology Co., Jinan, China) recorded the continuous accelerometer and gyroscope output. The complete sensor assembly was housed in a protective ABS enclosure, measuring 136 (length) mm × 71 (width) mm × 56 (height) mm and weighing 308 g. Figure 1 presents the full hardware schematic and the circuit diagram.

#### 2.1.3. Experimental Protocol

Prior to the experimental trials, each participant viewed a pre-recorded instructional video that thoroughly demonstrated the correct procedures for every experimental task. The custom-built IMU module was secured to the participant’s waist using a rigid mounting cradle and multiple fastening belts to minimize any relative motion between the sensor and the body. It was positioned over the left anterior iliac crest [42]. Prior studies indicate that placing a single IMU at the waist, with the sensor axes broadly aligned to the anatomical anterior–posterior, medial-lateral, and proximal-distal directions, yields more consistent signals for postural analysis [54] and highly accurate fall detection compared with other body locations [55]. A main reason is that the waist is located close to the body’s center of mass. This positioning allows the recorded dynamics to capture global balance loss with minimal interference from limb-specific motions. Consequently, numerous studies have reported strong pre-impact fall detection performance using a single waist-mounted IMU [35,40,41,42]. Furthermore, it enhances comfort and reduces the rate of complaints [40]. During the experiments, participants wore all required safety gear, including helmets, goggles, gloves, as well as knee and elbow pads. To facilitate data collection, a workstation PC equipped with a Bluetooth receiver was situated approximately 2 m from the testing zone.

**Figure 2:**
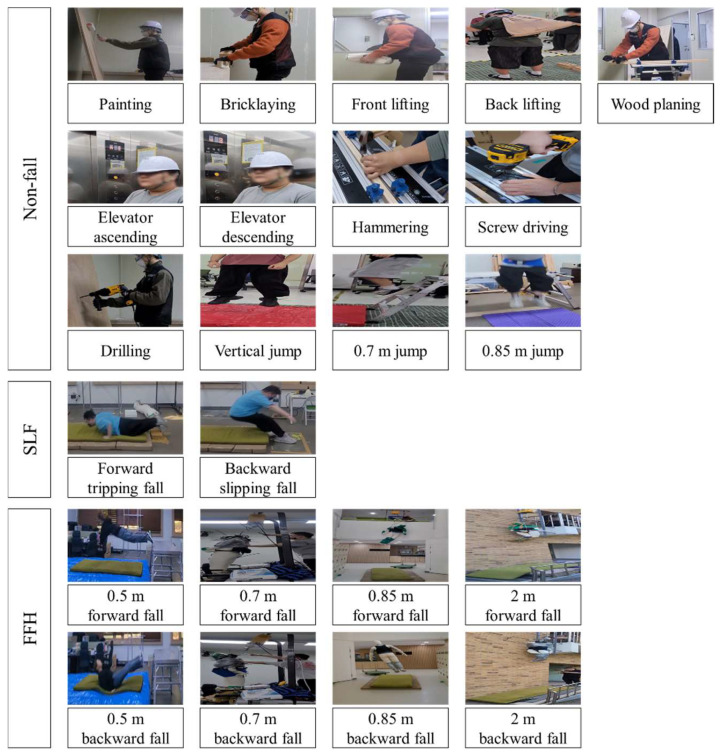
Non-fall, SLF, and FFH data acquisition experiments involved thirteen construction-site non-fall activities, such as high-vibration tool operation and jumping. Representative SLF scenarios included trip and slip falls. Typical FFH scenarios were conducted from heights of up to 2 m, with all falls from heights above 0.5 m performed using a validated human dummy.

Participants were directed to realistically perform a variety of non-fall construction-related activities, as well as SLF and FFH scenarios. A total of thirteen construction-site-specific non-fall activities, displayed in Figure 2, were chosen according to findings from prior research in construction activity recognition and fall detection: painting, bricklaying, front lifting, back lifting, wood planing, elevator ascending, elevator descending, hammering, screw driving, drilling, vertical jumping, jumping from 0.7 m, and jumping from 0.85 m [38,39,56,57,58,59]. Prior to each activity, participants maintained a stationary position for approximately 3 s to ensure accurate IMU sensor calibration, and then executed the assigned task following the instructional video. Each activity concluded with an additional 3 s period of standing still. Activities such as painting, bricklaying, front and back lifting, wood planing, elevator ascending and descending, hammering, screw driving, and drilling were each performed for about 5 s per trial, which constituted a single, complete data sample. For the vertical jump task, participants were asked to jump vertically from the ground using their natural effort level. Jumps from 0.7 m and 0.85 m heights were conducted using a ladder with adjustable height; a layer of high-density ethylene foam was positioned at the landing area to reduce injury risk. SLF trials required participants to simulate forward tripping and backward slipping falls, as depicted in Figure 2, according to established protocols from earlier studies [35,40,42]. These simulated falls were performed safely on gymnasium mats combined with high-density ethylene foam for impact absorption. The FFH tests included forward and backward falls from different heights: 0.5 m, 0.7 m, 0.85 m, and 2 m [31,38,39], as illustrated in Figure 2. Self-performed falls from 0.5 m were conducted safely onto an inflatable safety mattress. To address the increased risk at greater heights (>0.5 m), experiments for these conditions were conducted using a human dummy (Madamade, Gyeonggi-do, South Korea), which is approximately 1.80 m tall and weighs about 10 kg, constructed of a soft but durable knit material. This dummy has undergone validation in previous relevant studies, which found no significant difference in IMU signal patterns compared to those of human subjects during forward FFHs [38,39]. The dummy was elevated to the designated heights (0.7 m, 0.85 m, and 2 m) by an electric mini winch (UH300-12, UDT, Daegu, Republic of Korea) and then dropped onto gymnasium mattresses. Furthermore, 16 variations in ADLs (standing, forward lying, backward lying, leftward lying, rightward lying, sitting, sit-to-stand, stand-to-sit, stand-to-lying, lying-to-stand, walking, bending while walking, jumping, transition from walking to jumping, ascending stairs, and descending stairs), 10 types of near-fall scenarios (forward trip near-fall, backward trip near-fall, forward slip near-fall, backward slip near-fall, forward misstep near-fall, near-side fall to the right, near-side fall to the left, forward near-fall during sit-to-stand, forward near-fall due to hit/bump, and backward near-fall due to hit/bump), and an additional 8 categories of SLFs (forward fall, backward fall, leftward fall, rightward fall, fall while standing, sitting on empty chair, forward fall while walking, backward fall while walking) were chosen and performed in accordance with the literature and previous studies [35,40,41,42]. Upon completion of all experiments, a comprehensive dataset was compiled, consisting of 5991 non-fall activity samples, 1630 SLF samples, and 729 FFH samples.

### 2.2. Proposed Prediction Method for Non-Fall, SLF, and FFH Events

**Figure 3:**
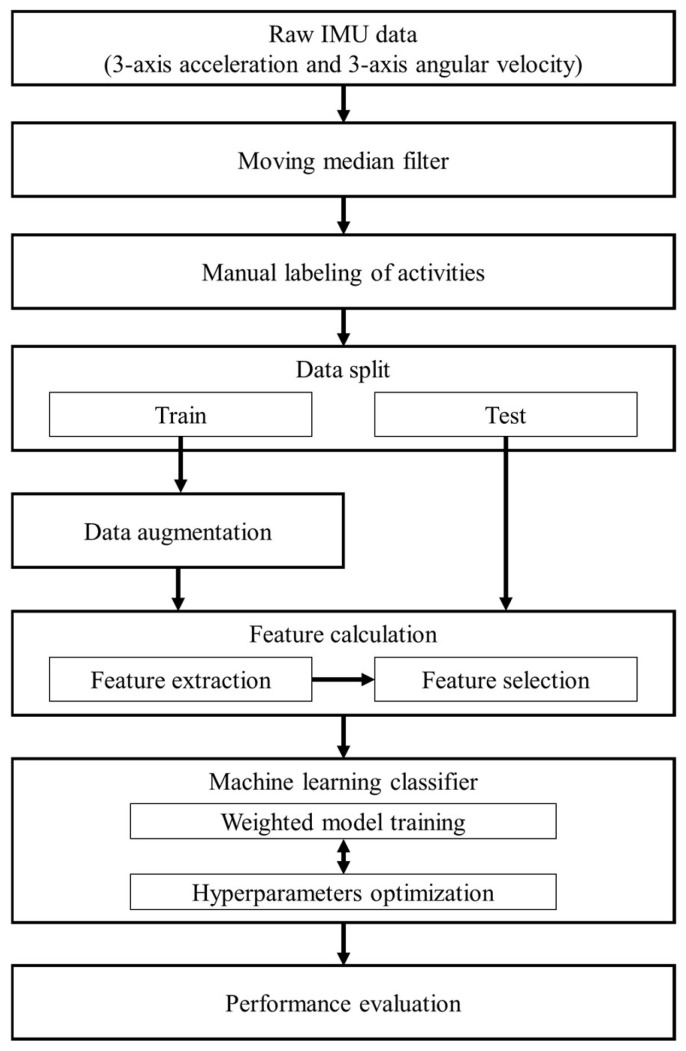
Flowchart of the proposed method to discriminate between non-fall, SLF, and FFH events.

The stepwise procedure for the proposed machine learning-based approach to differentiate among non-fall, SLF, and FFH events is depicted in Figure 3. First, we cleaned the raw tri-axial accelerometer and gyroscope signals by removing corrupted or incomplete records. The signals were then segmented into fixed-length windows, each containing 40 samples. To suppress high-frequency noise while preserving important dynamics, we applied a moving median filter to denoise each window. We manually labeled every window using the accelerometer’s sum magnitude vector (SMV) in conjunction with time-synchronized video, marking non-fall segments that corresponded to various construction activities, as well as the pre-impact portions of SLFs and FFHs. Next, we split the labeled data into training and test sets using stratified sampling to ensure that class proportions, particularly the minority fall classes, were preserved in both partitions. Data augmentation was applied only to the training subset to enhance variability and generalization. Feature construction occurred in two stages: first, we computed time-series descriptors from each window; then, we applied statistical aggregation to create compact features that highlight class-specific patterns. An ensemble feature-selection step was conducted to retain the most informative variables for the multiclass classification task. Using these features, we trained a class-weighted machine-learning classifier with hyperparameter optimization to determine the best model configuration. Finally, we evaluated the classifier’s performance on the held-out test set to obtain an unbiased estimate of accuracy and generalizability.

#### 2.2.1. Data Pre-Processing and Labeling

**Figure 4:**
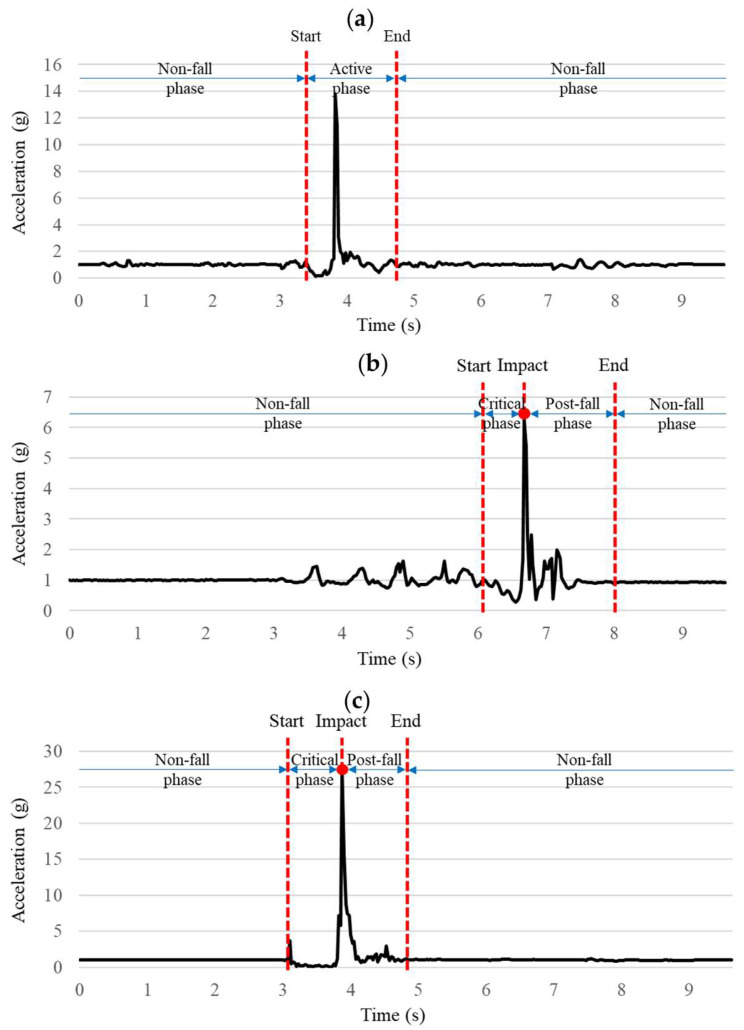
Acceleration SMV signal comparison between (**a**) stand to jump from a 0.85 m height, (**b**) walking to trip forward ground fall, and (**c**) stand to forward ground fall from a 2 m height.

All collected IMU sensor data were initially scrutinized for duplicate entries, data integrity issues, and potential errors arising during experimental recording; any corrupted or incomplete records were excluded from subsequent analysis. Each validated sensor data sample was then segmented into fixed-length windows comprising 40 data points. A moving median filter was subsequently employed on each window to attenuate high-frequency noise while retaining essential signal features. Manual labeling was performed by jointly inspecting the SMV computed from the tri-axial accelerometer and the time-synchronized video recorded with a GoPro Hero camera (San Mateo, CA, USA). Participants remained stationary for about 3 s before and after each trial to provide clear synchronization markers between the IMU and the video, which served as reference data for labeling and verification. Data from these stationary (non-active) intervals were excluded from all recorded samples. For non-fall activities, the extracted windows captured only the active motion segment, as illustrated in Figure 4a. This segment was determined by visually observing variations in the SMV signal following the 3 s stationary interval, with reference to the video. Similarly, for SLF and FFH events, each IMU recording was partitioned into three distinct phases (Figure 4b,c): the non-fall phase encompassing routine, stable movements preceding the fall; the critical phase, defined from the moment of balance loss to ground impact; and the post-fall phase, during which the subject remains motionless until coming to a complete rest after impact. Specifically, Figure 4b presents a representative SLF signal, with the critical phase beginning at the moment of loss of balance and ending at impact. Similarly, Figure 4c displays a representative FFH signal, where the critical phase encompasses the longer free-fall interval between the loss of balance and impact. Given the focus of this study on pre-impact detection of SLF and FFH events, only the critical phase data windows, representing periods before impact, were extracted and labeled accordingly. Consequently, the resulting manually labeled dataset consisted of segments clearly marked as non-fall activities, and critical-phase segments pertaining to SLF and FFH events, thereby supporting accurate training of a multiclassification algorithm. This fully labeled dataset was stratified into separate training and test subsets; to promote class balance and improve model robustness, data augmentation—including jittering, scaling, and temporal warping at four intensity levels [41]—was applied exclusively to the training set, while the test data remained unaltered.

#### 2.2.2. Two-Stage Feature Extraction

**Figure 5:**
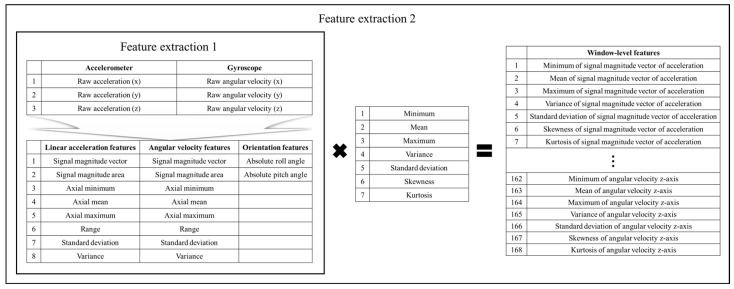
Two-stage IMU feature extraction pipeline.

In this study, a two-stage feature extraction methodology is presented to obtain meaningful and computationally efficient features from raw IMU signals, specifically designed to differentiate between non-fall activities, SLF, and FFH events. As shown in Figure 5, the first step focuses on calculating time-series features directly from the IMU sensor’s tri-axial accelerometer and angular velocity data. For each sensor window, core features including SMV, signal magnitude area (SMA), minimum, mean, maximum, maximum-minimum difference, standard deviation, variance, roll, and pitch angles are determined. These particular features have shown substantial effectiveness and reliability in prior works on IMU-based fall detection and activity recognition, and crucially, can be rapidly computed on wearable devices with limited memory and processing capabilities [40,60,61]. However, a limitation of relying solely on a stage-1 approach, as seen in related studies, is that computing data along the tri-axial accelerometer and gyroscope axes within a window treats the window as quasi-stationary. This method under-represents the temporal evolution within the window and fails to capture transitional or rhythm-dependent motions [62,63]. Therefore, in the next stage, statistical aggregation is applied at the window level to better summarize and boost the distinctiveness of the features derived from stage one. For every extracted time-series feature, seven descriptive statistics—minimum, mean, maximum, variance, standard deviation, skewness, and kurtosis—are computed over the entire window, yielding a robust set of statistical features [40,64]. The same set of statistical descriptors was computed for both the features obtained from the first stage and, separately, for the raw tri-axial accelerometer and angular velocity signals. This approach further expanded the feature set and captured vital motion properties. However, using only stage-2 temporal features computed over time for each axis, as was performed in previous studies, captures cadence and dynamic patterns that are critical for distinguishing activities with similar static statistics. By considering the axes largely independently, this method may overlook important inter-axis relationships and directionality information [62,65]. Unlike the previously used single-stage methods, the proposed two-stage scheme—first extracting inter-axis descriptors and then summarizing their temporal evolution—integrates spatial and temporal evidence into a compact representation. This two-step extraction method efficiently condenses the raw sensor data into a highly informative 168-dimensional feature vector for each window, supporting efficient downstream machine learning tasks. Moreover, the feature and statistic selections are rooted in both demonstrated effectiveness and computational low-cost, ensuring compatibility with the practical limitations of wearable system implementations [64].

#### 2.2.3. Ensemble Feature Selection

**Figure 6:**
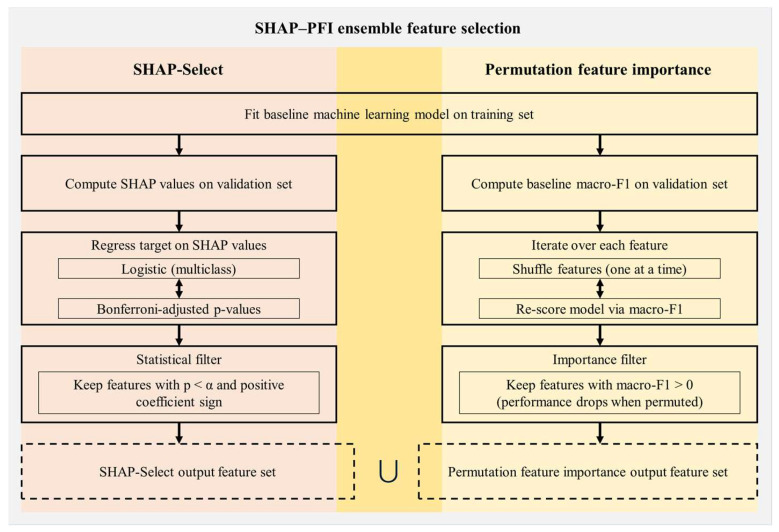
Proposed ensemble SHAP-PFI feature selection.

This study introduces an ensemble-based hybrid feature selection approach that integrates state-of-the-art SHAP-Select [48] with permutation feature importance (PFI) methods [49] to systematically determine the most relevant and influential features for classifying non-fall activities, SLF events, and FFH events, as shown in Figure 6. As an initial step, a baseline weighted XGBoost classifier was constructed using all 168 extracted features, and its hyperparameters were optimized through the Bayesian optimization method [66] to maximize macro-F1 score on the validation set.

The first phase of our approach utilized SHAP-Select, an advanced hybrid technique that combines game-theoretic model interpretation with statistical inference to identify significant predictors [48]. Using the trained weighted XGBoost model, we initially calculated SHAP (SHapley Additive exPlanations) values for each of the 168 features based on the validation set. These values, derived from cooperative game theory, measure the marginal contribution of every feature to individual predictions [67]. Next, to determine statistical significance, a multinomial logistic regression was performed with the true class labels as the dependent variable and the SHAP values of all features as independent variables. A predictor was selected only if its coefficient showed a positive association and remained statistically significant after a strict Bonferroni correction for multiple comparisons (*p* < 0.05) [48,68]. This process ensures that the retained features consistently exhibit a positive and statistically robust effect on model predictions. Although offering both computational efficiency and interpretability, this approach exhibits some recognized shortcomings relevant to our task. In datasets with class imbalance, the unweighted logistic regression portion may be heavily influenced by the majority class, resulting in features important for minority class prediction (SLFs and FFHs) being excluded due to insufficient statistical power [69,70]. In addition, substantial collinearity among features can attenuate individual SHAP values, leading to the inadvertent removal of useful, yet correlated, predictors [71].

To overcome these limitations, the second phase implemented PFI as a complementary, performance-focused validation method [49]. PFI functions by empirically evaluating the effect of an individual feature on the model’s predictive accuracy. It accomplishes this by systematically permuting (i.e., randomly shuffling) the values of a single feature within the validation set, effectively disrupting its association with the target variable, and measuring the corresponding reduction in model performance. Importantly, employing the weighted machine learning model with the macro-F1 score as the metric, PFI accords equal significance to each class, ensuring recognition of features essential for the minority SLF and FFH classes when their alteration results in a marked accuracy loss. As PFI does not assume any specific relationship or complexity in a feature’s influence, it is capable of identifying features that are strong negative indicators or exhibit complex non-linear or interaction effects, which might be overlooked by the SHAP-Select approach [72]. Nevertheless, a drawback of PFI is its propensity to undervalue the significance of highly correlated features, since permuting one may cause little change in performance if another redundant feature compensates [72,73]. This particular limitation is mitigated to some extent by SHAP-Select, which offers a model-internal analysis less susceptible to the permutation-associated biases characteristic of PFI.

The final stage of our methodology entailed forming the definitive feature set by taking the union of those features identified by both SHAP-Select and PFI. This ensemble strategy provides a robust, dual-validation framework by combining two distinct approaches to determining feature importance, yielding a more thorough and dependable feature subset. It integrates the model-focused explanatory capacity of SHAP-Select, which pinpoints features with statistically validated and consistent contributions, with the performance-driven, empirical assessment of PFI, which substantiates the direct effect of features on predictive accuracy. This methodology acts as a safeguard: PFI can recover empirically vital features that may not meet the rigorous statistical thresholds of SHAP-Select due to issues like class imbalance or collinearity, whereas SHAP-Select retains features that demonstrate consistent contributions but might be omitted by PFI due to redundancy. Consequently, the feature set produced by this ensemble selection method is not only robust and interpretable, but also better insulated against the inherent biases of any one selection technique, thus enhancing confidence in its appropriateness for the complex, imbalanced, multi-class classification challenge addressed.

#### 2.2.4. Weighted Machine Learning Models

In this study, gradient boosting algorithms were chosen as the most suitable machine learning models for classification involving compact wearable devices, based on their proven performance, computational efficiency, and applicability to embedded systems [74,75,76]. Gradient boosting approaches natively process tabular numeric features without extensive preprocessing and maintain high predictive accuracy even with the limitations of wearable sensor devices [77]. Specifically, advanced gradient boosting frameworks [50] such as XGBoost [74], LightGBM [75], and CatBoost [76] were adopted.

Hyperparameter optimization was carried out using Bayesian optimization, with each model (XGBoost, LightGBM, and CatBoost) tuned independently. The optimal hyperparameters for XGBoost comprised learning rate, number of boosting iterations, maximum tree depth, sub-sample ratio of training rows, column sampling ratio per tree, minimum loss reduction to split, minimum child weight, and L1/L2 regularization coefficients. For LightGBM, additional hyperparameters included maximum number of leaves. The hyperparameters optimized specifically for CatBoost comprised learning rate, number of boosting iterations, maximum tree depth, column sampling ratio per tree, bagging temperature and L2 leaf regularization. The hyperparameter search aimed to maximize the macro-F1 score on the validation set.

Furthermore, due to the significant class imbalance (with non-fall activities far exceeding SLF and FFH events), sample weighting was utilized during training to reduce the model’s bias toward the majority class. Each sample weight ($w_{i}$) was calculated using the inverse-frequency approach, thereby ensuring fair representation of each class during model optimization [70,78]:

(1) $$w_{i}=\;\frac{N}{K\times \;n_{c(i)}}$$

In this context, N indicates the total number of training samples, K designates the total number of classes, and $n_{c(i)}$ specifies the number of samples in class c(i). This weighting technique allows balanced influence from both minority and majority classes, directly mitigating issues caused by skewed data distributions.

 The selection of these models is supported by contemporary literature that evidences their strong performance and computational efficiency in embedded and wearable applications, where resource limitations are commonly encountered [77,79]. Gradient boosting methods, such as those adopted here, produce models that are both compact and interpretable, enabling real-time application on low-power devices and meeting the operational requirements of wearable health monitoring systems [79,80].

#### 2.2.5. Performance Measure

In this study, the stratified 5-fold cross-validation (CV) approach was utilized to rigorously assess the performance of the classification models. Stratified CV ensures that each fold retains class distributions comparable to those in the original dataset, which helps to address potential biases arising from class imbalance and allows for a reliable estimation of the model’s generalizability. An ablation analysis was first carried out to examine the effectiveness of the proposed ensemble feature selection technique by benchmarking its results against features individually chosen by SHAP-Select and PFI. The macro F1-score served as the main evaluation metric due to its equal consideration of each class distribution, making it especially relevant for datasets characterized by class imbalance. The macro F1-score is defined as:

(2) $${F1}_{\mathrm{macro}}=\frac{1}{K}\sum_{k=1}^{K}\frac{2\cdot TP_{k}}{2\cdot TP_{k}+FP_{k}+FN_{k}}$$

where $TP_{k}$, $FP_{k}$, and $FN_{k}$ represent true positives, false positives, and false negatives for class k, and K is the total number of classes.

To further investigate the discriminative capability of the proposed features, a comparative evaluation was performed with IMU feature sets derived from previous related studies [35,39] utilizing Uniform Manifold Approximation and Projection (UMAP) [81]. UMAP enables the visualization of high-dimensional data by projecting it into a lower-dimensional space while maintaining the integrity of both local and global data structures. Well-separated and distinct clusters observed in UMAP embeddings indicate features that are more likely to support effective multi-class classification. Furthermore, we assessed the classification performance of the selected features via the optimized Conv-LSTM algorithm as documented in recent studies [39], and compared results based on our proposed feature set with those using previously established IMU features from recent works [35,39]. Conv-LSTM was specifically implemented for this comparison because of its demonstrated ability to effectively model spatiotemporal dependencies typical in sequential sensor data, making it particularly suitable for multi-class classification tasks that involve IMU signals.

To conduct a thorough performance assessment and comparative study of state-of-the-art boosting algorithms (XGBoost, LightGBM, and CatBoost), several evaluation metrics were utilized, including macro accuracy, macro sensitivity, macro specificity, macro Matthews correlation coefficient (MCC), and class-calculated Precision-Recall Area Under Curve (PR-AUC). In addition to the previously discussed macro F1-score, these metrics are defined using $TP_{k}$, $TN_{k}$, $FP_{k}$, and $FN_{k}$, corresponding to true positives, true negatives, false positives, and false negatives for class k, respectively. Here, K indicates the number of classes in the dataset. The definitions of the employed metrics are as follows:

(1) Macro accuracy, which evaluates the average classification accuracy across all classes.

(3) $${Accuracy}_{macro}=\frac{1}{K}\sum_{k=1}^{K}\frac{TP_{k}+TN_{k}}{TP_{k}+TN_{k}+FP_{k}+FN_{k}}$$

(2) Macro sensitivity, which quantifies the model’s capacity to correctly detect positive cases in each class.

(4) $${Sensitivity}_{macro}=\frac{1}{K}\sum_{k=1}^{K}\frac{TP_{k}}{TP_{k}+FN_{k}}$$

(3) Macro specificity, indicating the ability of the model to identify negative samples accurately across classes.

(5) $${Specificity}_{macro}=\frac{1}{K}\sum_{k=1}^{K}\frac{TN_{k}}{TN_{k}+FP_{k}}$$

(4) Macro MCC, selected for its insensitivity to class imbalance and comprehensive integration of all confusion matrix terms.

(6) $${MCC}_{macro}=\frac{1}{K}\sum_{k=1}^{K}\frac{{TP}_{k}\cdot {TN}_{k}-{FP}_{k}\cdot {FN}_{k}}{\sqrt{\left({TP}_{k}+{FP}_{k}\right)\left({TP}_{k}+{FN}_{k}\right)\left({TN}_{k}+{FP}_{k}\right)\left({TN}_{k}+{FN}_{k}\right)}}$$

(5) Class-calculated PR-AUC, providing a robust assessment of classification performance under class imbalance by quantifying the precision-recall trade-off.

Statistical significance across the evaluated models was analyzed using Analysis of Variance (ANOVA) and followed by Tukey’s Honestly Significant Difference (HSD) post hoc testing, applying a significance criterion of *p* < 0.05.

Additionally, computational efficiency indicators—specifically average training time, inference time, and system latency—were evaluated. These measures were included to determine the practical deployment potential of the models for real-time operation on resource-constrained wearable platforms, which is essential for timely event detection in safety-critical domains.

The complete evaluation protocol was implemented using Python 3.11.11 (Python Software Foundation, Wilmington, DE, USA). Statistical analyses were performed in IBM SPSS Statistics (SPSS 29.0, IBM Corporation, Armonk, NY, USA). All computations utilized a workstation featuring a 16-core Intel(R) Xeon(R) Gold 6226R processor (2.90 GHz) (Intel Corporation, Santa Clara, CA, USA), an NVIDIA RTX A5000 GPU (24 GB VRAM)(NVIDIA Corporation, Santa Clara, CA, USA), and 256 GB RAM, operating on Windows 10.

## 3. Results and Discussion

### 3.1. Hyperparameter Optimization Results

Table 1 outlines the hyperparameter search domains, types, value ranges, and the finalized hyperparameters determined via Bayesian optimization for the XGBoost, LightGBM, and CatBoost algorithms.

The optimized learning rates for each algorithm fell within a moderate-to-low range: XGBoost at 0.298, LightGBM at 0.020, and CatBoost at 0.092. This aligns with comparative study indicating that small step sizes offer a favorable accuracy-generalization trade-off [50]. Such moderate learning rates enhance model stability by striking a balance between convergence speed and generalization ability. For ensemble sizes, the optimization process produced relatively compact models with moderate numbers of boosting iterations (XGBoost: 254 iterations, LightGBM: 760 iterations, CatBoost: 539 iterations), demonstrating that ensembles of this size were sufficient to reach high classification accuracy. These configurations are advantageous for efficient computational execution. The tree complexity parameters selected by optimization also indicated moderate complexity: XGBoost and CatBoost utilized maximum depths of 9 and 8, respectively, while LightGBM chose 203 leaves and a maximum tree depth of 10. This level of complexity shows that IMU-derived feature patterns were effectively represented without excessively complex models, potentially lowering computational requirements during inference. Subsampling parameters converged at relatively high values for row subsampling (XGBoost: 0.959, LightGBM: 0.892) and for column subsampling (XGBoost: 0.948, LightGBM: 0.382, CatBoost: 0.892), supporting an effective compromise between the robustness imparted by stochastic sampling and reliable retention of critical discriminative information. In addition, moderate values were selected for regularization hyperparameters. For example, XGBoost determined a minimum loss reduction of 0.036 along with L1 and L2 regularization coefficients of 0.006 and 1.13 × 10^−5^, respectively. LightGBM set its minimum split gain at 0.919, with L1 and L2 coefficients of 0.030 and 7.47 × 10^−4^, respectively. CatBoost selected a bagging temperature of 0.657 and an L2 leaf regularization value of 1.146. Collectively, these regularization parameters demonstrate appropriate control over model complexity, which helps prevent overfitting while maintaining strong predictive power. In summary, the hyperparameter optimization performed in this study revealed unambiguous and consistent patterns: moderate learning rates, ensembles of moderate size, balanced tree complexities, and deliberately managed regularization.

### 3.2. Comparative Analysis of Feature Selection Methods

**Figure 7:**
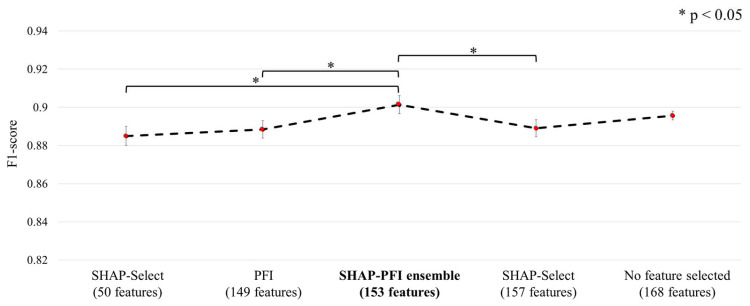
Five-fold CV macro-F1 performance of baseline XGBoost models trained with different feature subsets: SHAP-Select (50 features), PFI (149), proposed SHAP-PFI ensemble (153), SHAP-Select (non-negative, 157), and all features (168). Subsets marked with asterisks (*) differ significantly from the SHAP-PFI ensemble according to a paired *t*-test (*p* < 0.05).

The comparative analysis outcomes for the proposed SHAP-PFI ensemble approach versus individual SHAP-Select, PFI methods and without selected features are illustrated in Figure 7.

The macro F1-score, computed as an average over 5-fold CV, was adopted as the performance metric in order to provide a balanced assessment, which is especially crucial due to the dataset’s inherent class imbalance. The proposed SHAP-PFI ensemble approach, selecting 153 features, achieved the highest macro F1-score of 0.901 ± 0.005. This result was slightly superior to the score obtained with all 168 features without selection (0.895 ± 0.002). More importantly, the ensemble strategy demonstrated clear outperformance over each stand-alone feature selection method: SHAP-Select with only key features (50 features, 0.885 ± 0.005) and those limited to non-negative SHAP values (157 features, 0.889 ± 0.005), as well as the PFI technique (149 features, 0.888 ± 0.005). Statistically significant differences confirmed the superior effectiveness of the ensemble method compared to these individual techniques. The small yet significant improvement provided by the SHAP-PFI ensemble relative to using the full feature set highlights the ensemble’s ability to isolate and preserve only the most salient and informative variables. Such targeted feature selection not only contributes to greater model robustness, but can also reduce computational demands without compromising, and in some cases enhancing, predictive accuracy.

A closer analysis of the comparative outcomes clarifies the ranking shown in Figure 7. The SHAP-Select method, which retained just the 50 statistically significant variables, yielded a macro F1-score of 0.885, indicating a decrease relative to the model with the entire feature set. The stringent Bonferroni correction effectively reduced type-I errors, but in doing so also excluded numerous correlated predictors that collectively capture nuanced differences between the predominant class of non-fall motions and the minority classes of SLFs and FFHs. This exclusion resulted in the loss of critical information, weakening sensitivity to important events. Extending the set to include the 157 variables with non-negative SHAP values allowed for some recovery of informative cues and improved the macro F1-score to 0.889, although this value still did not reach the performance of the complete feature set. Nevertheless, this broader inclusion also reintroduced a number of lower-impact features whose contributions varied across individuals, limiting the overall improvement.

The permutation feature importance method retained 149 variables and produced a macro F1-score of 0.888. Permutation testing evaluates the empirical impact on total accuracy; however, in the case of multicollinearity, it frequently underestimates the contribution of any variable among a highly correlated set, since the shared information remains among the other features. As a result, certain moderately informative yet redundant variables were excluded, reducing performance and leading to a macro F1-score that failed to surpass the score achieved with the full set of features.

The ensemble subset amalgamates all variables that are either statistically robust or empirically crucial, while simultaneously eliminating those deemed both insignificant and neutral to performance. This systematic selection yielded 153 features and achieved the highest macro F1-score of 0.901. Relative to the comprehensive 168-feature baseline, the ensemble omits 15 redundant features, resulting in reduced model variance, enhanced generalization to previously unseen site activities, and decreased durations for both training and inference. These findings substantiate that a dual-criterion approach can address the singular shortcomings of either significance-driven or permutation-driven filters, thus maintaining minority-class information without compromising computational efficiency.

### 3.3. Comparative Analysis of the Discriminative Capability of Proposed and Previous IMU Feature Sets

**Figure 8:**
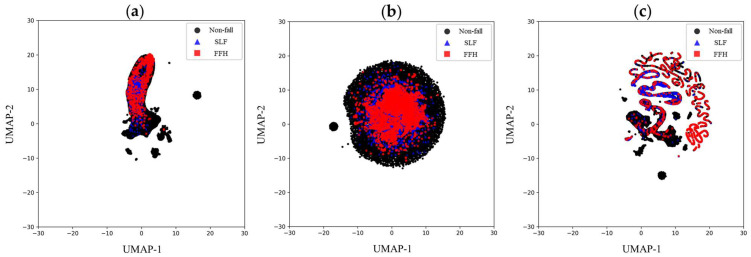
UMAP visualizations comparing class separability using IMU-derived feature sets: (**a**) Eight IMU-derived features proposed by Lee et al. [39]; (**b**) Thirty IMU-derived features including accelerometer and orientation-related features from a preceding study [35]; (**c**) Proposed 153 IMU-derived features selected by the SHAP-PFI ensemble method. Class labels: non-fall (●, black circles), SLF (▲, blue triangles), and FFH (■, red squares) events.

To facilitate a visual assessment of the discriminative capability of the ensemble-selected features, Figure 8 provides a side-by-side comparison between the feature set identified in this research and those suggested in previous studies [35,39].

The UMAP embedding generated from Lee et al.’s feature set (Figure 8a) shows significant overlap and poorly defined boundaries among non-fall, SLF, and FFH events, indicating limited ability to distinguish between these categories. This overlap indicates challenges in accurately differentiating falls from non-fall activities, which may undermine detection performance. In a similar manner, the UMAP embedding for the previously proposed 30 feature set (Figure 8b) illustrates densely packed data points with pronounced class overlap. Although orientation-related features are included, this embedding shows that such features, even when supplemented by basic statistical attributes, are insufficient for distinguishing between the key fall types (SLFs and FFHs) and non-fall activities, indicating a lack of ability to capture unique kinematic markers associated with each class. In contrast, the UMAP plot for the proposed 153 features (Figure 8c) displays a more distinct and organized clustering arrangement. Non-fall activities are represented by clear and separated clusters, while SLF and FFH groupings exhibit well-defined structures with substantially less overlap than earlier feature sets. This more clear class separation demonstrates the capability of the ensemble-based hybrid feature selection method to extract relevant and discriminative IMU-derived features. The side-by-side results in Figure 8 indicate that the proposed SHAP-PFI ensemble approach effectively identifies a feature subset that increases the discernibility of SLF and FFH events from non-fall activities. However, complete class separation was not fully achieved: some overlap remains due to extreme, highly dynamic construction movements that produce kinematic patterns partially resembling those of SLFs and FFHs. As a result, further improvements in classification accuracy with the present feature set will necessitate employing advanced learning models capable of capturing complex, non-linear relationships among features and robustly handling severe class imbalance.

**Figure 9:**
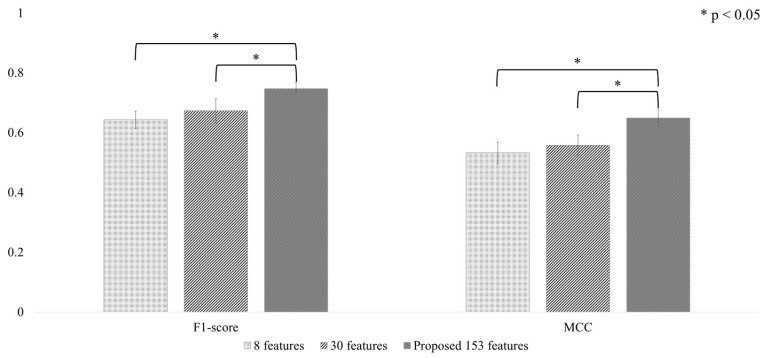
Comparison of performance analysis results for the optimized Conv-LSTM model trained using different IMU-derived feature sets: 8 features as defined by Lee et al. [39], 30 features from our previous research [35], and 153 features selected by the SHAP-PFI ensemble method introduced in this study. Here, asterisks (*) indicate statistically significant differences (paired *t*-test, *p* < 0.05) relative to the proposed 153-feature set.

The comparative evaluation of the optimized Conv-LSTM algorithm [39] utilizing three distinct IMU-derived feature sets—8 basic features as defined by Lee et al., 30 features identified in our previous research, and the 153 features selected by the SHAP-PFI ensemble in this study—is depicted in Figure 9. The assessment of performance was conducted using two metrics: macro F1-score and MCC, with results averaged across 5-fold CV.

The Conv-LSTM model attained the highest macro F1-score of 0.747 ± 0.002 and macro MCC of 0.649 ± 0.003 when trained with the proposed set of 153 features. This model configuration demonstrated significantly better results than the version trained on the 30-feature set, achieving roughly 11% higher macro F1-score (0.673 ± 0.040) and an improvement of about 17% in macro MCC (0.557 ± 0.034), with both differences reaching statistical significance (paired t-test, *p* < 0.05). Additionally, when compared to the 8-feature set, the model achieved an approximately 16% increase in macro F1-score (0.643 ± 0.029) and a 22% improvement in MCC (0.532 ± 0.036). These notable and consistent improvements highlight the superior discriminative potential offered by carefully selecting a larger set of features.

Nonetheless, although the expansion and optimization of the feature space led to clear performance improvements for the Conv-LSTM model, it should be emphasized that the optimized Conv-LSTM algorithm still exhibited substantially lower performance compared to the optimized baseline XGBoost algorithm when both utilized the identical set of 153 features, with the XGBoost model achieving an average macro F1-score of 0.901. This performance gap primarily arises from intrinsic algorithmic differences in handling tabular, sensor-derived datasets. Specifically, gradient-boosting decision tree algorithms are well-suited for processing structured, tabular data characterized by heterogeneous feature distributions and limited training samples—typical scenarios in sensor-based research—without the need for extensive hyperparameter adjustment or elaborate model architecture [82,83]. By contrast, the Conv-LSTM model, although highly effective for sequential data, generally demands substantially larger and more balanced datasets to adequately train its many parameters, which increases the risk of overfitting with smaller, heterogeneous sensor-based datasets, as demonstrated in previous wearable sensor classification literature [84,85]. Accordingly, these findings underscore the practical benefit of using gradient boosting-based algorithms for reliable and resource-efficient SLF and FFH detection with wearable IMU sensor data.

### 3.4. Comparison of Boosting Models Performance

Table 2 presents the classification performance of the three state-of-the-art boosting models—XGBoost, LightGBM, and CatBoost—assessed across 8 distinct metrics following 5-fold CV. Statistical analysis was performed using one-way ANOVA and Tukey’s HSD post hoc tests (*p* < 0.05), facilitating a robust evaluation of performance differences among the models.

XGBoost achieved the highest macro accuracy (0.985 ± 0.001), closely followed by LightGBM (0.984 ± 0.001); both models significantly outperformed CatBoost (0.976 ± 0.001; F = 139.317, *p* < 0.001). The same pattern held for macro F1-score and MCC, with XGBoost (macro F1-score of 0.901, macro MCC of 0.869) and LightGBM (macro F1-score of 0.897, macro MCC of 0.864) ahead of CatBoost’s outcomes (macro F1-score of 0.860, macro MCC of 0.811). This outcome aligns with their learning principles. XGBoost constructs trees level-wise using second-order optimization and incorporates explicit L1 and L2 regularization, which is effective for moderate, well-engineered numeric features. In contrast, LightGBM employs histogram binning and efficient leaf-wise growth to identify precise thresholds under appropriate regularization. These characteristics, highlighted in prior studies [50,74,75], are well-suited to our two-stage numeric IMU features and address the moderate class imbalance.

In contrast, CatBoost showed significantly greater macro sensitivity (0.907 ± 0.008) and macro specificity (0.962 ± 0.002), exceeding the performances of both XGBoost (macro sensitivity of 0.881 and macro specificity of 0.949) and LightGBM (macro sensitivity of 0.890 and macro specificity of 0.954). These differences were statistically significant (macro sensitivity: F = 20.732, *p* < 0.001; macro specificity: F = 39.958, *p* < 0.001). Although CatBoost’s strengths in handling categorical features are not directly utilized in our fully numerical feature set, its ordered boosting—which reduces prediction shift and helps curb overfitting in small or imbalanced datasets—likely improved recall on rare events. This advantage is crucial for wearable fall detection, where minimizing false negatives is essential [39].

All three models produced equivalent and high PR-AUC values (0.999) for the prevalent non-fall class, with no statistically significant difference (*p* = 0.478). However, performance diverged for fall-specific classes (SLFs and FFHs), with XGBoost and LightGBM yielding higher scores than CatBoost. For SLFs, XGBoost (0.953) and LightGBM (0.951) noticeably surpassed CatBoost (0.937). A weaker but similar pattern was present in FFHs, where XGBoost and LightGBM (0.871 and 0.872) showed modest advantages over CatBoost (0.856). The results indicate that XGBoost and LightGBM provide a strong balance between precision and recall for the minority fall classes, particularly when the feature set captures diverse inter-axis kinematics and temporal dynamics from the IMU signals.

Overall, the results demonstrate a trade-off in performance: XGBoost and LightGBM achieve superior aggregate metrics (macro accuracy, macro F1-score, macro MCC, PR-AUC), whereas CatBoost outperforms in macro sensitivity and specificity. For scenarios that prioritize maximizing SLF and FFH detection coverage, CatBoost’s greater sensitivity may offer advantages. Nevertheless, for real-time embedded systems that demand low false-alarm rates and high computational efficiency, the consistently higher macro F1-score and macro MCC of XGBoost and LightGBM make them more suitable choices. Notably, LightGBM delivered detection quality similar to XGBoost with marginally improved macro specificity, suggesting a potentially optimal compromise between detection accuracy and generalization.

Table 3 presents the average run-time performance of the three boosting-based detectors, including training time, inference time, and window-level latency, as measured by 5-fold CV.

The models exhibit substantial variation in training durations. XGBoost completes training in 43,621 ms, LightGBM in 136,783 ms, and CatBoost in 208,539 ms. These differences arise from the ensemble sizes chosen for each model—254, 760, and 539 trees, respectively—and the distinct learning strategies involved. XGBoost utilizes a level-wise histogram approach that requires fewer data scans. In contrast, LightGBM implements a leaf-wise algorithm, and CatBoost uses ordered boosting, both of which encompass larger search spaces and therefore prolong training duration. Although models are trained off-device, reduced build times facilitate faster recalibration during field deployment.

During real-time use, inference time and latency are critical to operational feasibility. Inference time quantifies the computation needed to score a complete test fold, and latency expresses this cost per window, indicating the response delay on the wearable device. XGBoost processes all windows in 22.06 ms, with a mean latency of 1.51 × 10^−3^ ms per window. CatBoost processes in 29.29 ms, corresponding to a latency of 2.01 × 10^−3^ ms, while LightGBM takes 218.97 ms, yielding a window-level latency of 1.50 × 10^−2^ ms. All latency values are significantly lower than the available lead time, confirming that the airbag can be triggered before impact. The additional timing margin provided by XGBoost and CatBoost could also accommodate supplementary on-device functions, such as wireless transmission or recording.

When these run-time outcomes are evaluated together with the classification metrics in Table 2, XGBoost emerges as the most well-rounded approach. It demonstrates the highest macro F1-score and macro MCC while achieving the lowest latency and the fastest training time. CatBoost is preferable where macro sensitivity and macro specificity are prioritized, in spite of its longer fitting phase and slightly increased latency. LightGBM attains accuracy levels comparable to XGBoost, but its tenfold higher latency suggests it is more appropriate for deployment on edge or cloud platforms rather than on battery-powered wearable devices.

In summary, the experiments confirm that utilizing a single waist-mounted IMU, in conjunction with the two-stage feature pipeline and the SHAP-PFI ensemble feature selector, enables reliable classification of non-falls, SLFs, and FFHs, achieving a macro F1-score above 0.90 and maintaining window-level latency in the low-millisecond domain. The 153-feature subset facilitated sharper class separation compared to previously used IMU features. Out of the three gradient-boosting techniques, XGBoost attained the top macro accuracy and macro MCC, while CatBoost delivered the best macro sensitivity and specificity. LightGBM, despite being the slowest model, still satisfied real-time application requirements by completing fold-level inference in 219 ms with window-level latency of 1.50 × 10^−2^ ms.

Employing a fixed 40-sample windowing method yields mean lead times of 402 ms for SLFs and 640 ms for FFHs. Both values exceed the 130 ms lead time threshold necessary for wearable airbag deployment. The greater variability observed with FFHs is due to the diverse drop heights applied during data collection, resulting in distinctly different free-fall durations. These lead times indicate that the proposed detector provides ample advance notice for airbag inflation in both SLF and FFH scenarios, which is particularly significant because SLFs involve the shortest free-fall interval. Additionally, XGBoost and CatBoost further extend the safety margin, allowing more time for wireless communication or data recording and processing on the same microcontroller. While training time is generally less critical post-deployment, it aligned with predictions based on ensemble complexity and training methodology: XGBoost reached convergence three to five times more rapidly than the alternatives, thus facilitating future system recalibration in operational environments.

The detector also overcomes a critical limitation of previous studies by directly incorporating high-vibration tool operation and various jumping activities—movements that often lead to false alarms in earlier machine learning-based approaches [31,38]. Despite this increased challenge, the system retains state-of-the-art accuracy using lightweight feature sets and a compact model suitable for deployment on low-power embedded systems. These attributes establish the framework as a promising foundation for the sensing component of advanced SLF and low-altitude FFH prevention solutions, including wearable airbag devices targeting the construction sector.

Several limitations persist. First, as the data were collected in a controlled environment, the dataset does not encompass the full range of postures, tool usage, or surface conditions present on construction sites. Despite the protocol including numerous actions that are challenging to classify, some relevant movements may have been omitted. The dataset also demonstrates a pronounced class imbalance favoring non-fall activities, and while data augmentation and class weighting were implemented, these methods only partially mitigated the issue. Future research should integrate a greater number of field recordings or employ realistic synthetic IMU sequences, particularly for SLFs and FFHs, to achieve a more balanced dataset. Increasing activity diversity and leveraging state-of-the-art generative methodologies [86] will further enhance the reliability of IMU-based pre-impact SLF and FFH detection for real-world construction scenarios.

Second, for safety in this study, we simulated part of the FFH scenarios using a 10 kg anthropomorphic dummy, which is lighter than a typical construction worker. Given the short free-fall durations considered (heights up to 2 m), the dominant pre-impact dynamics captured by the IMU—such as sudden loss of support, near-ballistic specific force, and characteristic changes in angular rate—are primarily influenced by gravity rather than body mass. Therefore, the key signatures used for detection are expected to be applicable across different masses [87]. Consistent with this expectation, prior construction-focused studies found no significant differences in the IMU acceleration and angular velocity patterns between human falls and a 10 kg dummy at comparable heights. This supports the reliability of dummy-based data for developing pre-impact detection models [38,39]. We recognize that a dummy cannot replicate active human responses, such as bracing or trunk flexion, which can affect orientation and posture. Future research should involve tests using higher-mass surrogates and safe human fall protocols across a wider range of heights. This will help confirm whether the learned thresholds and lead times remain consistent across different body sizes and fall strategies, and allow for refinement of the model if systematic differences are identified.

Third, environmental conditions commonly found on construction sites—such as temperature fluctuations, high humidity, and strong winds—can degrade IMU accuracy. Changes in temperature and humidity can alter the dynamic response of MEMS accelerometers, affecting bias and scale and reducing effective bandwidth. Additionally, wind gusts can induce vibrations in the mounting, which may manifest as spurious inertial signals [88,89,90]. In this study, we partially mitigated these effects by housing the sensor in a protective ABS enclosure and securing it at the waist in a rigid cradle with multiple fastening belts to minimize relative motion. Additionally, we applied a moving-median filter to attenuate high-frequency noise while preserving salient features. Future work will incorporate environment-aware algorithms to further enhance the system. Specifically, we plan to implement learned temperature-drift compensation and calibration based on on-board temperature and humidity readings [88,89], and to adopt adaptive filtering methods—such as the Kalman filter—to suppress wind-induced vibration and resonance artifacts [90]. These additions are expected to enhance measurement reliability in harsh site conditions.

Finally, this study focused on developing a machine-learning framework for the pre-impact detection of SLFs and FFHs using a single waist-mounted IMU. Full end-to-end integration with a fall protection device was outside the scope of this research and should be addressed in future work. For deployment, a wearable airbag can provide effective protection against SLFs and FFHs, including falls from heights of up to approximately 5 m, when its design is tailored to the dominant injury patterns [24,25,26,27,28,29,30,31,32]. SLFs more often result in lateral impacts to the hip [40], whereas FFHs more often involve higher-energy contacts to the head and spine [12,15]. Therefore, the airbag should prioritize coverage of the hips, back, and head. It is essential to implement a fast inflation mechanism and optimize the gas volume to reduce impact loads in both SLFs and FFHs, while ensuring the total inflation time is shorter than the pre-impact lead time observed in our detections. Additionally, practical deployment on construction sites will require a focus on comfort and secure attachment, allowing the sensing device and wearable airbag to be worn reliably during routine tasks.

## 4. Conclusions

This study introduces a single-sensor, waist-worn IMU integrated device designed to identify pre-impact SLF and FFH events typical in construction environments. The employment of a two-step feature extractor, in conjunction with an ensemble SHAP-PFI feature selector, reduced 168 initial descriptors to 153 distinctive and informative features, yielding distinct class separation and an average 5-fold macro F1-score exceeding 0.90. Out of three fine-tuned boosting algorithms, XGBoost achieved the optimal compromise between accuracy (macro accuracy of 0.985), robustness (macro MCC of 0.869), and computational efficiency, performing per-window inference in 1.51 × 10^−3^—which is well within the average lead times of 402 ms (SLFs) and 640 ms (FFHs). CatBoost demonstrated the peak macro sensitivity and macro specificity, whereas LightGBM matched the accuracy but incurred higher inference delay. All evaluated models satisfied both the stringent detection accuracy and real-time processing requirements for wearable airbag actuation, even under high-vibration activities and jumps often leading to false alerts. The minimized feature set, low resource usage, and exceptional classification effectiveness establish the proposed machine learning-based detector as a viable sensing foundation for advanced risk assessment and new-generation fall-prevention solutions in complex construction settings.

## Figures and Tables

**Table 1:** Hyperparameters optimization results.

Hyper-Parameter	Data Type	Search Range	XGBoost	LightGBM	CatBoost
Learning rate	float, log-uniform	[0.005–0.30]	0.298	0.020	0.092
Number of boosting iterations	integer, uniform	[100–800]	254	760	539
Maximum tree depth	integer, uniform	[3–10]	9	10	8
Sub-sample ratio of training rows	float, uniform	[0.50–1.00]	0.959	0.894	—
Column sampling ratio per tree	float, uniform	[0.30–1.00]	0.948	0.382	0.892
Minimum loss-reduction to split	float, uniform	[0–5]	0.036	0.919	—
Minimum child weight	(XGB) integer, uniform	[1–10]	4	0.038	—
	(LGB) float, log-uniform	[1 × 10^−3^–10]			
Maximum number of leaves	integer, uniform	[15–255]	—	203	—
Bagging temperature	float, uniform	[0–1]	—	—	0.657
L2 leaf regularization	float, log-uniform	[1 × 10^−3^–10]	—	—	1.146
L1 regularization coefficient	float, log-uniform	[1 × 10^−6^–10]	0.006	0.030	—
L2 regularization coefficient	float, log-uniform	[1 × 10^−6^–10]	1.13 × 10^−5^	7.47 × 10^−4^	—

**Table 2:** Comparative results of the optimized boosting models (XGBoost, LightGBM, CatBoost).

	XGBoost (1)	LightGBM (2)	CatBoost (3)	ANOVA Results	Post Hoc Test
**Accuracy**	0.985 ± 0.001	0.984 ± 0.001	0.976 ± 0.001	F = 139.317, *p* = 0.000	(1), (2) > (3)
**Sensitivity**	0.881 ± 0.005	0.890 ± 0.007	0.907 ± 0.008	F = 20.732, *p* = 0.000	(3) > (2), (1)
**Specificity**	0.949 ± 0.001	0.954 ± 0.003	0.962 ± 0.002	F = 39.958, *p* = 0.000	(3) > (2) > (1)
**F1-Score**	0.901 ± 0.005	0.897 ± 0.005	0.860 ± 0.007	F = 82.651, *p* = 0.000	(1), (2) > (3)
**MCC**	0.869 ± 0.005	0.864 ± 0.006	0.811 ± 0.009	F = 118.898, *p* = 0.000	(1), (2) > (3)
**PR-AUC** **(Non-fall)**	0.999 ± 0.000	0.999 ± 0.000	0.999 ± 0.000	F = 0.784, *p* = 0.478	(1), (2), (3)
**PR-AUC** **(SLFs)**	0.953 ± 0.003	0.951 ± 0.002	0.937 ± 0.005	F = 30.585, *p* = 0.000	(1), (2) > (3)
**PR-AUC** **(FFHs)**	0.871 ± 0.004	0.872 ± 0.006	0.856 ± 0.010	F = 8.633, *p* = 0.005	(2), (1) > (3)

**Table 3:** Run-time results of the optimized boosting models.

	Train Time [ms]	Inference Time [ms per Fold]	System Latency [ms per Window]
XGboost	43,621	22.061	1.51 × 10^−3^
LightGBM	136,783	218.965	1.50 × 10^−2^
CatBoost	208,539	29.288	2.01 × 10^−3^

## Data Availability

The data that support the findings of this study are available on request from the corresponding author. The data are not publicly available due to privacy or ethical restrictions.
